# Sclerostin and Cardiovascular Risk: Evaluating the Cardiovascular Safety of Romosozumab in Osteoporosis Treatment

**DOI:** 10.3390/biomedicines12122880

**Published:** 2024-12-18

**Authors:** Shi-Hsun Chiu, Wen-Tien Wu, Ting-Kuo Yao, Cheng-Huan Peng, Kuang-Ting Yeh

**Affiliations:** 1 School of Medicine, Tzu Chi University, Hualien 970, Taiwan; 108311103@gms.tcu.edu.tw (S.-H.C.); timwu@tzuchi.com.tw (W.-T.W.); peng0913@tzuchi.com.tw (C.-H.P.); 2 Department of Orthopedics, Hualien Tzu Chi Hospital, Buddhist Tzu Chi Medical Foundation, Hualien 970, Taiwan; tayao0318@tzuchi.com.tw; 3 Institute of Medical Sciences, Tzu Chi University, Hualien 970, Taiwan; 4 Graduate Institute of Clinical Pharmacy, Tzu Chi University, Hualien 970, Taiwan

**Keywords:** sclerostin inhibition, romosozumab, osteoporosis, cardiovascular risk, bone mineral density

## Abstract

**Background/Objectives:** Osteoporosis and cardiovascular disease (CVD) share common risk factors and pathophysiological mechanisms, raising concerns about the cardiovascular implications of sclerostin inhibition. Romosozumab, a monoclonal antibody that targets sclerostin, is effective in increasing bone mineral density (BMD) and reducing fracture risk. However, evidence suggests that sclerostin inhibition may adversely affect vascular calcification, potentially increasing the risk of myocardial infarction (MI) and stroke. **Methods:** This review synthesizes data from clinical trials, such as ARCH, BRIDGE, and FRAME, alongside genetic studies and observational analyses, to evaluate the cardiovascular safety of romosozumab. PubMed was searched for relevant studies published within the last five years. Studies addressing the relationship between romosozumab and cardiovascular outcomes were included, emphasizing both its efficacy in osteoporosis management and potential cardiovascular risks. **Results:** Romosozumab significantly improves BMD and reduces fracture risk in postmenopausal women and men with osteoporosis. However, clinical trials report an increased incidence of major adverse cardiovascular events (MACE), particularly in patients with pre-existing cardiovascular conditions such as chronic kidney disease (CKD), diabetes, or prior CVD. Genetic studies indicate that *SOST* gene variants may also influence cardiovascular outcomes. **Conclusions:** While romosozumab is an effective treatment for osteoporosis, careful cardiovascular risk assessment is crucial before initiating therapy, especially for high-risk populations. Long-term studies are needed to evaluate chronic safety. Future therapeutic strategies should aim to maintain bone health while minimizing cardiovascular risks, ensuring a balance between efficacy and safety in osteoporosis treatment.

## 1. Introduction

Osteoporosis, which is characterized by skeletal fragility and the deterioration of the bone microarchitecture, is associated with an increased risk of fractures, particularly among postmenopausal women. Postmenopausal women experience declines in bone mineral density (BMD) and alterations in bone quality [1]. With the global population aging, the incidence of fractures has markedly increased, resulting in an increasing burden on healthcare systems worldwide [2]. The management of osteoporosis is further complicated by substantial overlaps in the risk factors and pathophysiological mechanisms between osteoporosis and cardiovascular disease (CVD), indicating a potential interplay between low BMD and increased cardiovascular risk [3]. Patients with osteoporosis often exhibit a higher likelihood of developing coronary artery disease and stroke, whereas those with CVD are at an increased risk of experiencing bone loss and fractures [4,5]. Common risk factors, such as advanced age, smoking, a sedentary lifestyle, and diabetes, contribute to both conditions, indicating a shared pathophysiological pathway [4]. Additionally, biochemical mediators, including osteoprotegerin, sclerostin, and *fibroblast growth factor 23* (*FGF-23*), play dual roles in bone and cardiovascular health, thereby adding complexity to the relationship between these diseases [4,5]. Sardu et al., in 2020, explored the molecular mechanisms linking inflammation to CVDs, highlighting emerging biomarkers such as sirtuins and microRNAs as critical diagnostic, prognostic, and therapeutic targets for managing CVD-related inflammation [6].

Studies have also identified other major contributory factors, such as elevated homocysteine levels, low-density lipoprotein (LDL), and total cholesterol, to osteoporosis in older patients, whereas high-density lipoprotein and body weight offer protective effects [6].

**Figure 1:**
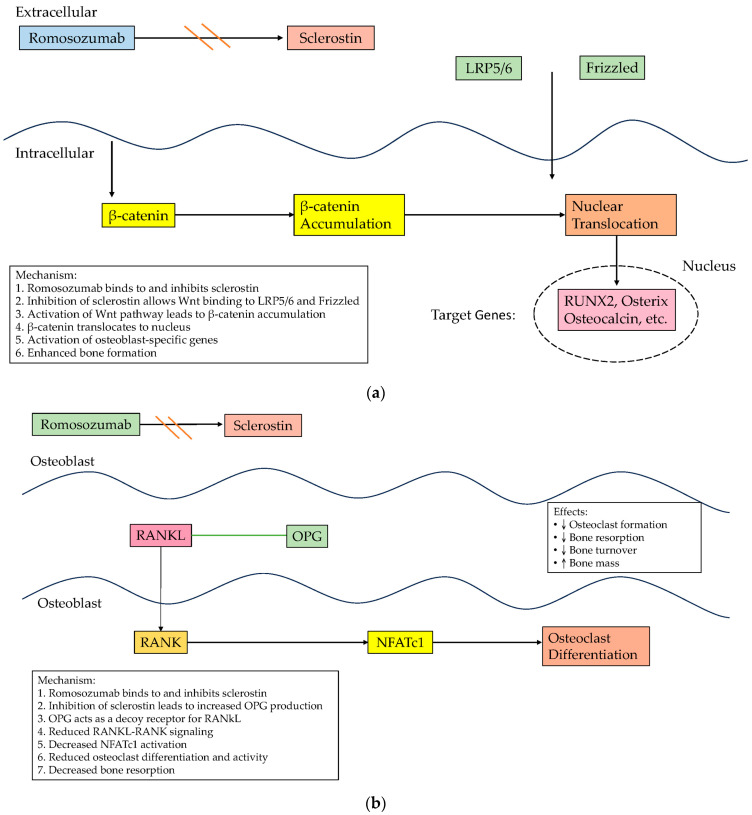
(**a**) Romosozumab’s mechanism of action in promoting bone formation; (**b**) Romosozumab’s mechanism in reducing bone resorption through its effects on osteoclasts. Double-orange-parallel-line symbol: inhibition.

At the molecular level, the Wnt signaling pathway is essential for osteoblastogenesis, the biological process responsible for bone formation mediated by osteoblasts [7]. The activation of this pathway promotes the differentiation, maturation, and survival of osteoblasts, thereby enhancing bone formation and contributing to overall bone health. The pathway is activated when a Wnt ligand binds to LDL receptor-related proteins 5 and 6 (Lrp5/6), triggering intracellular signaling cascades that lead to the nuclear translocation of β-catenin, which promotes the expression of the gene-encoding proteins involved in osteoblastogenesis. Disruptions in the Wnt pathway consequently contribute to the development of osteoporosis. Sclerostin, a glycoprotein secreted by osteocytes, serves as a critical extracellular antagonist of the Wnt signaling pathway. By binding to the Lrp5/6 coreceptors, sclerostin inhibits the interaction between Wnt ligands and their receptors, thereby suppressing bone formation. Elevated levels of sclerostin can hinder osteoblast activity, thus facilitating the progression of osteoporosis. In this context, targeting sclerostin is a promising therapeutic strategy for osteoporosis. Romosozumab, a monoclonal antibody specifically designed to inhibit sclerostin, has demonstrated dual efficacy in promoting bone formation and reducing bone resorption (Figure 1). Clinical trials have demonstrated that postmenopausal women treated with romosozumab exhibited a reduced risk of vertebral and clinical fractures, thereby supporting its clinical utility [8,9]. However, despite the evident benefits of romosozumab for bone health, concerns persist regarding the potential cardiovascular risks associated with its use, particularly due to its mechanism of action [10]. Although the inhibition of sclerostin effectively enhances bone density, it may also exert unintended effects on cardiovascular tissues, potentially increasing the risk of CVD [11]. Katsanos et al., in 2017, identified the B-type natriuretic peptide as a key predictor of 1-year mortality in elderly patients undergoing hip fracture surgery, emphasizing the prognostic value of pre- and postoperative cardiac biomarkers to predict clinical outcomes in patients with bone loss or fractures [12]. Given the dual role of sclerostin in the regulation of both bone and cardiovascular health, conducting thorough assessments of the potential cardiovascular implications associated with sclerostin inhibition is crucial.

This review critically evaluated the current evidence regarding the cardiovascular risks associated with sclerostin inhibition, with a particular focus on the role of romosozumab in postmenopausal women with low bone mass. By examining the dual impact of sclerostin on both bone and cardiovascular health, this review provided a comprehensive overview of the benefits and risks associated with romosozumab therapy, offering valuable insights for future research and clinical practice [10].

## 2. Literature Search Method for Review

The literature search for this review was conducted on 7 December 2024 by using PubMed as the primary database. The search strategy was meticulously designed to encompass all relevant studies related to the effect of sclerostin inhibitors, particularly romosozumab, on cardiovascular health. The selected search terms were intended to address both the specific intervention and its broader cardiovascular implications. To ensure a focused and comprehensive selection, the search included the following keywords in the article titles: ((((sclerostin [Title/Abstract]) OR (romosozumab [Title/Abstract])) OR (Evenity [Title/Abstract]))) AND (((cardiovascular [Title]) OR (hypertension [Title])) OR (artery [Title])). Filters were applied to restrict the results to English-language articles published within the past 5 years. The Boolean operators AND and OR were used to optimize the query and effectively capture the desired scope of research.

The following studies were included in this review:

- studies that specifically addressed the effects of sclerostin or its inhibitor, romosozumab, on cardiovascular health;
- studies that employed either observational or interventional designs and were published in peer-reviewed journals within the past 5 years;
- high-quality systemic reviews and meta-analyses that synthesized relevant primary research and contributed to understanding broader trends or confirming key findings;
- commentaries on other articles that introduced novel perspectives or offered unique interpretations relevant to the cardiovascular implications of sclerostin inhibitors;
- studies were restricted to those published in English to ensure consistency and feasibility in the analysis.

These criteria were established to ensure that the review included only studies that reflect current clinical practices and provided up-to-date evidence relevant to cardiovascular outcomes in patients receiving romosozumab.

Furthermore, specific exclusion criteria were established to ensure the relevance and quality of the review. Articles were excluded if they met any of the following criteria:

- did not directly address the effects of sclerostin or romosozumab on cardiovascular health;
- were commentaries on other articles without introducing novel perspectives;
- featured author responses to comments that did not offer new insights.

This selection process was designed to ensure the inclusion of high-quality primary research, insightful commentaries, and comprehensive secondary analyses that represented original and relevant findings. To facilitate structured data collection, a standardized data extraction form was employed. This form recorded essential details, including the authors, year of publication, study design, sample size, key findings, and conclusions. This systematic approach enabled an efficient synthesis of the literature, thereby supporting the critical evaluation of the impacts of sclerostin inhibitors on cardiovascular outcomes.

## 3. Sclerostin and Cardiovascular Risk Factors

Several studies have demonstrated a strong association between elevated sclerostin levels and cardiovascular risk across various patient populations. Sclerostin, a glycoprotein that inhibits the Wnt/β-catenin signaling pathway, has been implicated in the exacerbation of cardiovascular risk factors [13,14,15,16,17,18,19,20,21,22,23,24,25,26,27,28,29]. For instance, in patients undergoing peritoneal dialysis, high serum sclerostin levels are associated with poorer survival outcomes and an increased risk of cardiovascular events [13]. Similarly, long-term hemodialysis (HD) patients with elevated sclerostin levels have exhibited increased cardiovascular mortality [14]. However, although some studies have indicated an association between sclerostin and coronary artery calcification, others have argued that sclerostin levels alone do not independently increase the risk of cardiovascular events or mortality [15]. A notable prospective cohort study revealed that high sclerostin levels were correlated with lower cumulative freedom from major cardiovascular events, such as myocardial infarction (MI), stroke, and hospitalizations due to heart failure, indicating an increased overall cardiovascular risk in high-risk populations, such as patients undergoing hemodialysis [16]. Increased circulating sclerostin levels have been associated with greater insulin resistance, fat mass accumulation, and impaired glucose metabolism, suggesting its dual role in both metabolic and cardiovascular dysfunction [17]. Moreover, elevated sclerostin levels have been consistently associated with vascular calcification, particularly in patients with chronic kidney disease (CKD) and diabetes, both of which are conditions that exacerbate cardiovascular risk [18,19,20,21]. González-Salvatierra et al. demonstrated that elevated circulating sclerostin levels correlate with increased cardiovascular risk, as assessed by the SCORE2-Diabetes algorithm [22]. Morena-Carrere et al. identified severe coronary artery calcifications (CAC) in CKD patients as a major predictor of adverse cardiovascular events, with elevated sclerostin and other bone-related markers exacerbating cardiovascular mortality [23].

Moreover, the inhibitory role of sclerostin on the Wnt/β-catenin signaling pathway, which is essential for vascular health, may contribute to the development of atherosclerotic plaques and adverse cardiac outcomes, such as left ventricular hypertrophy and pulmonary hypertension [24,25,26,27]. Elevated sclerostin levels have been associated with structural changes in the heart, including an increase in left ventricular diameter, a reduction in the ejection fraction, and an increase in pulmonary artery pressure, further underscoring the significant cardiovascular impact of sclerostin [28]. In patients with CKD, increased sclerostin production—rather than decreased renal clearance—has been identified as the primary cause of its elevated levels, highlighting the complex regulation of sclerostin in this population [28]. Similar findings have been reported in studies involving patients referred for coronary angiography. Although no direct correlation was observed between sclerostin levels and coronary artery disease (CAD) severity, higher sclerostin levels were significantly associated with higher levels of hs-CRP, Klotho protein, BMI, and a lower glomerular filtration rate, suggesting a multifaceted role in both cardiovascular and bone health [29]. The reviewed studies are summarized in Table 1.

## 4. Sclerostin and Cardioprotective Role

In contrast to its association with cardiovascular risk, emerging evidence suggests that sclerostin may exhibit cardioprotective effects under certain conditions. In patients with CAD, higher sclerostin levels have been correlated with less severe vascular calcification and improved vascular health, indicating a potential protective role against the progression of atherosclerosis [30]. Genetic studies have supported this perspective, demonstrating that individuals with genetically elevated sclerostin levels exhibited a lower incidence of CAD and other cardiovascular conditions [30]. Notably, although elevated serum sclerostin levels have been linked to severe cardiovascular events, some researchers have proposed that these elevated levels may serve as a compensatory mechanism to protect cardiovascular health. Mendelian randomization studies have reinforced this hypothesis, indicating that higher genetically determined sclerostin levels are associated with a reduced risk of MI, hypertension, DM, coronary artery calcification, coronary artery disease, and worsened cardiovascular biomarkers, including reduced HDL cholesterol and elevated triglyceride levels [31,32]. For example, one study revealed that a reduction in sclerostin levels was associated with an increased risk of MI, type 2 diabetes, and coronary artery calcification, whereas lower sclerostin levels were correlated with a higher risk of MI and hypertension [31].

In patients with CKD, higher serum sclerostin levels are associated with more severe carotid artery atherosclerosis, which may represent a compensatory response aimed at limiting vascular calcification through the inhibition of the Wnt/β-catenin pathway [33,34]. Moreover, sclerostin has emerged as a critical marker of cardiovascular risk in CKD–mineral and bone disorder (CKD-MBD). Its regulatory role extends beyond the skeleton, influencing vascular remodeling, coronary artery calcification, and left ventricular hypertrophy through complex interactions with mineral metabolism and pro-inflammatory pathways [35]. Similarly, in patients with type 2 diabetes, elevated sclerostin levels were demonstrated to be correlated with increased carotid intima-media thickness (cIMT), potentially as a protective mechanism to slow the progression of atherosclerosis [36]. Its anti-calcification effects have been shown to play a role in modulating chronic inflammation linked to cardiometabolic disorders, such as type 2 diabetes [37].

Other studies have highlighted broader cardiovascular benefits across various age groups. For example, in pediatric patients with primary hypertension, elevated sclerostin levels are linked with lower systolic blood pressure, whereas in postmenopausal women with nonobstructive CAD, higher sclerostin levels help preserve coronary function despite vascular changes [38,39]. Additionally, in older patients undergoing percutaneous coronary intervention, elevated sclerostin levels were associated with improved survival rates and fewer adverse cardiovascular events, which may be attributed to its role in mitigating the progression of atherosclerosis [31,40]. These findings indicate that sclerostin plays a complex role in cardiovascular health, exerting protective effects on specific patient populations. Future research should investigate therapeutic strategies that retain sclerostin’s cardioprotective properties and actions while promoting bone formation. Studies targeting loop 3 of sclerostin offer promising avenues for next-generation therapies that balance these dual functions [41]. The findings from the reviewed studies are summarized in Table 2.

## 5. Sclerostin Inhibitors and Cardiovascular Events

**Figure 2:**
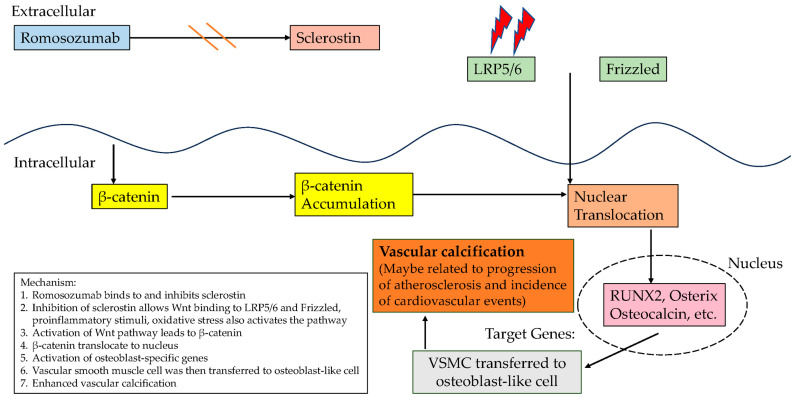
Romosozumab’s potential influence on the cardiovascular system through sclerostin-related pathway. Double-orange-parallel-line symbol: inhibition; double-lightening symbol: stimulation.

Romosozumab, a monoclonal antibody that inhibits sclerostin, was first approved for clinical use in Japan in 2019 for the treatment of high-fracture-risk osteoporosis [42]. Although romosozumab has demonstrated efficacy in increasing BMD and reducing fracture risk, concerns persist regarding its cardiovascular effects [43]. Nonclinical safety evaluations have yielded inconsistent results, with animal studies indicating neutral effects on atherosclerosis, whereas human studies report inconclusive findings, underscoring the need for thorough cardiovascular risk assessments before prescribing romosozumab [44,45]. Several clinical trials have raised concerns about an increased incidence of cardiovascular events, such as MI and stroke, in patients receiving romosozumab [46,47]. The drug’s impact on the Wnt/β-catenin pathway, which is critical for both bone and cardiovascular health, may contribute to vascular calcification [48]. This concern is particularly relevant for patients with CKD, in whom romosozumab may exacerbate cardiovascular problems by promoting vascular calcification (Figure 2). However, the exact mechanisms remain unclear and warrant further investigation [49]. Notably, post-marketing surveillance in Japan indicated a higher incidence of MACE among romosozumab users, which was attributed to differences in regulatory policies and prescribing practices, especially in older patients with pre-existing cardiovascular risks [50].

A systematic review and Bayesian network meta-analysis suggested that romosozumab ranked higher in cardiovascular risk compared to placebo, though the findings were limited by methodological differences among the included trials [51]. A systematic review and meta-analysis revealed no significant cardiovascular association with denosumab, another antiresorptive drug, but indicated that romosozumab might increase the risk of major adverse cardiovascular events (4P MACE), including cardiovascular death, MI, stroke, and heart failure [52]. Although some researchers have argued that the cardiovascular risks associated with romosozumab may have been overstated due to the methodological limitations in these studies, others have contended that further investigation is necessary to clarify these risks [52,53,54].

In response to growing concerns, Bovijn et al. performed a meta-analysis of three randomized controlled trials (BRIDGE, ARCH, and FRAME) and performed a Mendelian randomization analysis by using bone mineral density-increasing alleles at the *SOST* locus. Their findings indicated that *SOST* variants may be associated with cardiovascular risks, such as MI and coronary revascularization [55]. Despite criticism from other researchers who argued that genetic confounders might have influenced these results, Bovijn and colleagues defended their conclusions, emphasizing the need for further investigation into the cardiovascular safety of sclerostin inhibition [56,57]. There were some researchers who suggested that there was no higher cardiovascular risk with romosozumab compared to osteoporosis drugs, including PTH analogs [58,59,60,61]. However, cardiovascular risk assessment is crucial before initiating romosozumab treatment [62]. The reviewed studies are summarized in Table 3.

## 6. Efficacy and Safety of Romosozumab in Men with Osteoporosis

The BRIDGE study, a pivotal randomized controlled trial that focused exclusively on men with osteoporosis, demonstrated that romosozumab significantly increased BMD, like its effects in postmenopausal women [63]. Although the efficacy of romosozumab in enhancing bone health and reducing fracture risk is well established, concerns regarding its cardiovascular safety have become increasingly pertinent, particularly due to the cardiovascular events reported in multiple studies, including the ARCH and FRAME trials [55]. In the BRIDGE trial, eight cardiovascular events (4.9%) were reported in the romosozumab group compared with two events (2.5%) in the placebo group, suggesting a potential increase in cardiovascular risk [63]. This finding reflects broader concerns regarding the impact of romosozumab on cardiovascular health, primarily due to its inhibition of sclerostin, a key regulator of both bone and vascular health. The inhibition of sclerostin may promote vascular calcification, a known risk factor for MI, stroke, and other cardiovascular events [48,49]. Romosozumab exerts its effects through the Wnt/β-catenin signaling pathway, which is crucial for bone formation but also plays a role in maintaining vascular integrity. Disruption of this pathway may inadvertently result in adverse cardiovascular outcomes, particularly in men with underlying conditions such as CKD, diabetes, or a history of CVD [33,49]. Additionally, genetic studies have associated *SOST* variants (encoding sclerostin) with increased risks of MI and coronary revascularization [55].

**Figure 3:**
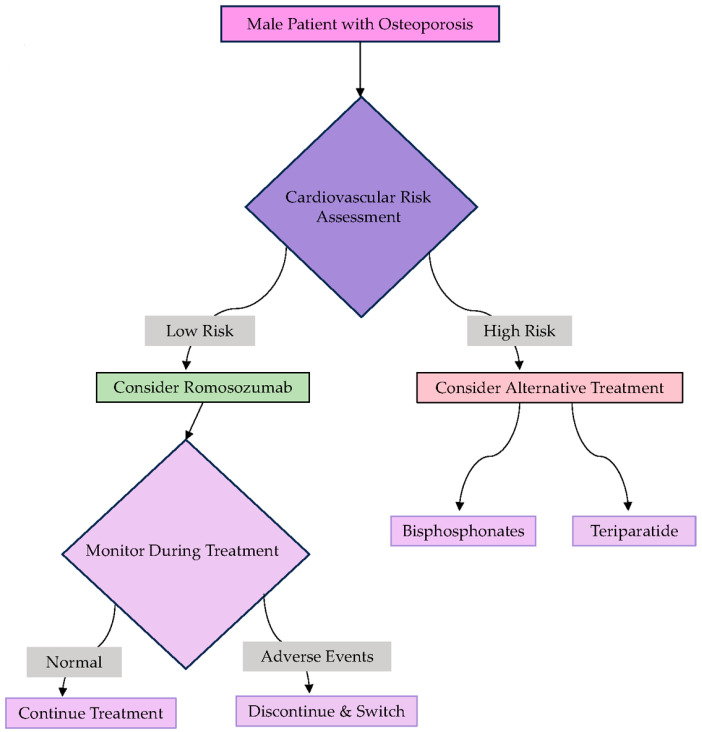
A clinical decision flowchart for men with osteoporosis.

Given these findings, a thorough assessment of cardiovascular risk is crucial when considering romosozumab for men, particularly those with preexisting cardiovascular risk factors. Regular monitoring during treatment, including ECG, lipid profiles, and blood pressure assessments, is crucial to mitigating potential cardiovascular complications. For men at high cardiovascular risk, alternative therapies may be more appropriate, and the decision to use romosozumab should be guided by a personalized risk–benefit analysis (Figure 3).

## 7. Pretreatment Assessments for Romosozumab Prescription

**Figure 4:**
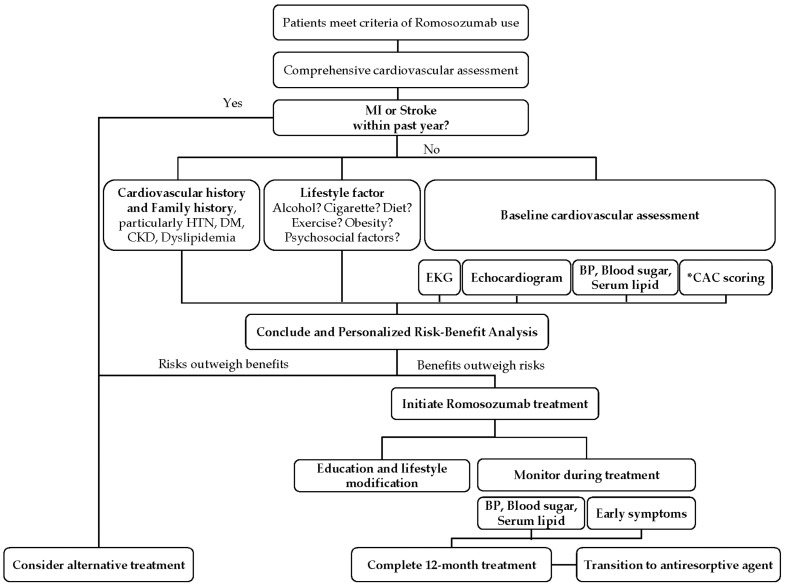
Our suggested screening flowchart for female osteoporotic patients treated with romosozumab. * Coronary artery calcium (CAC) scoring > 300 as the severe grade.

Before initiating romosozumab therapy, a comprehensive cardiovascular assessment is essential, particularly for patients at an elevated risk of cardiovascular events (Figure 4). Although the efficacy of romosozumab for improving BMD and reducing fracture risk is well established, its potential to increase the risk of MI and stroke underscores the need for meticulous pretreatment evaluation [46,47].

### 7.1. Cardiovascular History and Risk Factors

Patients with a recent history of MI, stroke, or other serious cardiovascular events should undergo careful assessment, as romosozumab has been associated with an increased risk of major cardiovascular events in clinical trials [42,55]. Additionally, the presence of preexisting conditions, such as hypertension, hyperlipidemia, and diabetes, or a history of CVD, warrants close examination, as these conditions may further elevate the risk of adverse events during romosozumab treatment [15,31].

### 7.2. Baseline Cardiovascular Assessments

A thorough baseline evaluation should include the following:

- electrocardiogram (ECG) and echocardiogram to detect any abnormalities in heart rhythm or structure;
- blood pressure measurements and lipid profile assessments to obtain insights into cardiovascular health;
- coronary artery calcium scoring for patients with preexisting cardiovascular risks to evaluate the extent of vascular calcification, which romosozumab may exacerbate by disrupting the Wnt/β-catenin pathway [48,49].

### 7.3. Monitoring During Treatment

Once romosozumab treatment is initiated, regular cardiovascular monitoring is essential. The following measures should be implemented:

- ongoing blood pressure monitoring to detect any signs of emerging hypertension;
- regular monitoring of glucose and lipid levels, particularly for patients with diabetes or metabolic conditions;
- surveillance for any signs of cardiovascular stress, such as chest pain, dizziness, or shortness of breath, with immediate follow-up if symptoms arise [38,44].

### 7.4. Lifestyle Modifications and Education

Patients should be encouraged to adopt heart-healthy lifestyle habits, including smoking cessation, regular exercise, and dietary changes to further mitigate cardiovascular risk. Patient education is critical for ensuring that individuals can recognize the early symptoms of cardiovascular issues and seek prompt medical attention if these symptoms arise [42].

### 7.5. Personalized Risk–Benefit Analysis

The decision to prescribe romosozumab must be based on an individualized risk–benefit analysis. Although romosozumab offers numerous advantages for patients with osteoporosis, its use should be approached with caution in individuals with a high cardiovascular risk. For patients at a low risk of CVD, romosozumab may serve as a valuable treatment option. However, for those at a higher risk, alternative treatment modalities should be considered.

## 8. Limitations and Future Directions

Despite the substantial advancements in elucidating the role of sclerostin and the efficacy of romosozumab in treating osteoporosis, several limitations persist in both the current research and clinical trials.

### 8.1. Study Design and Duration

A primary limitation of the current clinical trials on romosozumab is the lack of long-term follow-up data. Most studies have focused on short-term outcomes, typically spanning 1–2 years, which may not be sufficient to capture the complete range of long-term cardiovascular risks associated with sclerostin inhibition. For example, although romosozumab has demonstrated considerable efficacy in increasing BMD and reducing fracture risk over short-term periods, its long-term impact on vascular calcification and cardiovascular events remains unclear [43,44]. Therefore, future studies should prioritize extended follow-up periods to comprehensively evaluate the chronic safety of romosozumab, particularly in relation to cardiovascular health.

### 8.2. Inclusion of Diverse Patient Populations

Another notable limitation is the underrepresentation of diverse patient populations in clinical trials. Most studies have included postmenopausal women or men with osteoporosis, but specific high-risk groups, such as those with CKD, diabetes, or established CVD, have been often excluded. These groups are at a higher risk of both osteoporosis and cardiovascular complications, and their exclusion limits the generalizability of the findings [18,49]. Future research should consider observational studies or registries that could provide real-world insights into cardiovascular outcomes among patients receiving romosozumab despite contraindications.

### 8.3. Cardiovascular Risk Assessment

Although some trials have raised concerns regarding romosozumab’s potential to increase the risk of MI and stroke, the precise mechanisms through which sclerostin inhibition contributes to these outcomes remain inadequately understood. Mendelian randomization studies and genetic analyses have suggested a potential association between *SOST* and an increased risk of cardiovascular events. However, the clinical relevance of these findings continues to be a topic of debate [55]. Additionally, most studies have relied on broad assessments of MACEs without specifically investigating more subtle cardiovascular changes, such as vascular calcification or structural alterations in the heart and blood vessels. This lack of granular data may result in early cardiovascular changes that could eventually lead to more serious outcomes being overlooked [48].

### 8.4. Publication Bias and Language Bias

Another notable concern in the literature is the potential publication bias in favor of studies that have reported positive outcomes regarding romosozumab’s efficacy in increasing BMD and reducing fracture risk. By contrast, studies that have highlighted its adverse cardiovascular effects may be underreported. Furthermore, the inherent language bias arising from restricting the literature review to studies published in English may result in the exclusion of valuable data from non-English-speaking regions. This bias may skew the overall understanding of romosozumab’s safety profile. To mitigate these biases, future reviews should incorporate studies published in multiple languages and from a wider range of geographical regions to provide a more balanced view.

### 8.5. Exploring Alternative Therapeutic Targets

Although romosozumab has been proven to be a promising treatment option for osteoporosis, concerns regarding its cardiovascular safety profile warrant the exploration of alternative therapeutic strategies that can enhance bone formation without posing significant cardiovascular risks. One area of ongoing research focuses on the targeting of loop 3 of sclerostin, which may allow for the preservation of its cardiovascular protective actions while simultaneously promoting bone health [41]. This approach holds promise for the development of next-generation osteoporosis treatments that could offer improved safety profiles.

### 8.6. Individualized Risk Stratification

A crucial future direction in the management of patients receiving romosozumab is the development of personalized treatment protocols based on individual risk stratification. Given the conflicting evidence regarding the cardiovascular outcomes associated with romosozumab, establishing comprehensive pretreatment screening tools that accurately predict which patients are at a higher risk for cardiovascular complications is imperative. This approach could involve the integration of genetic markers, biomarkers of vascular health, and detailed cardiovascular risk assessments. By incorporating these elements, clinicians can make informed decisions about the appropriateness of romosozumab treatment [15,31]. Furthermore, personalized medicine approaches may optimize the therapeutic benefits of romosozumab while minimizing the potential harm.

### 8.7. Future Research Directions

To address the existing knowledge gaps and enhance the therapeutic profile of romosozumab, future studies should focus on the following key areas:

- Long-term cardiovascular safety of romosozumab in diverse populations, particularly those with preexisting cardiovascular conditions. The current body of evidence on romosozumab is limited by short follow-up durations in clinical trials, typically 1–2 years. This timeframe may be insufficient to fully assess long-term cardiovascular outcomes, such as major adverse cardiovascular events (MACE), heart failure, or progressive vascular calcification. Future randomized controlled trials (RCTs) should involve extended follow-up periods of 5–10 years to capture chronic cardiovascular risks;
- Mechanistic studies to elucidate the impact of sclerostin inhibition on the Wnt/β-catenin pathway in both bone and vascular tissues. Advanced mechanistic studies using preclinical models, such as genetically modified animals, organ-on-chip systems, and three-dimensional vascular tissue cultures, could elucidate these molecular interactions. Techniques like CRISPR-Cas9 genome editing and transcriptomic profiling could identify the specific signaling nodes linking sclerostin inhibition to both beneficial and adverse cardiovascular effects;
- Development of novel therapies that retain the bone-building benefits of romosozumab while minimizing the associated cardiovascular risks. Given the cardiovascular concerns linked to romosozumab due to its inhibition of sclerostin, future research should focus on selectively targeting specific structural domains of sclerostin. The study by Yu et al. [41] highlights that sclerostin’s loop 3 plays a central role in inhibiting bone formation while being non-essential for cardiovascular protection [41]. Genetic truncation and pharmacological inhibition of loop3 maintained sclerostin’s cardiovascular protective effects while promoting bone formation;
- Creation of personalized risk assessment models that integrate genetic, metabolic, and cardiovascular markers to optimize treatment outcomes. Future research should involve patient-centered models that incorporate quality-of-life assessments and long-term treatment satisfaction. Real-world data from patient-reported outcomes could inform shared decision-making processes, balancing osteoporosis treatment benefits with potential cardiovascular risks.

## 9. Conclusions

Our comprehensive analysis of sclerostin biology and romosozumab therapy has revealed critical insights into the complex relationship between bone metabolism and cardiovascular health. Sclerostin demonstrates a remarkable biological duality—while serving as a negative regulator of bone formation, it exhibits both detrimental and protective effects in the cardiovascular system. This dual nature is particularly evident in chronic kidney disease patients, where elevated sclerostin levels correlate with increased cardiovascular events, yet simultaneously show potential cardioprotective properties through the regulation of vascular calcification. Romosozumab, as a therapeutic intervention, presents a nuanced clinical profile that demands careful consideration. While it stands as a potent stimulator of bone formation, delivering significant improvements in bone mineral density and substantial reductions in fracture risk, its cardiovascular implications cannot be overlooked. The therapy’s association with increased cardiovascular events in high-risk populations, particularly those with pre-existing cardiovascular disease or chronic kidney disease, necessitates a thorough risk assessment and careful patient selection. Looking forward, the field must advance along three critical paths. First, therapeutic innovation should focus on developing more targeted approaches, such as loop 3-specific inhibition, that maintain cardiovascular safety while promoting bone formation. Second, comprehensive pre-treatment screening protocols must be refined and standardized to better identify at-risk patients. Finally, the implementation of personalized medicine approaches, tailoring treatment decisions to individual cardiovascular risk profiles, will be crucial for optimizing patient outcomes. This intricate balance between efficacy and safety underscores the importance of continued research and vigilant clinical practice in the evolving landscape of osteoporosis treatment.

## Figures and Tables

**Table 1:** Sclerostin and cardiovascular risk factors.

Author (Year)	Methodology	Study Characteristics and Sample Size According to Gender	Key Finding
Zou et al., 2020 [13]	POCS	165 dialysis patients (55.8% male, mean age: 56.5 years)	Positive association between sclerostin levels and cardiovascular events in peritoneal dialysis patients, not in hemodialysis patients.
Kalousová et al., 2019 [14]	POCS	106 long-term hemodialysis patients (60.34% male, mean age: 61 years)	Sclerostin levels predict cardiovascular mortality in long-term hemodialysis patients.
Ge et al., 2022 [15]	POCS	65 dialysis patients (HD: 26, PD: 39, 52.3% male, mean age: 48.4 years)	Although elevated sclerostin levels are associated with coronary artery calcification, they are not independently linked to a greater incidence of cardiovascular events and mortality.
Stavrinou et al., 2020 [16]	POCS	80 hemodialysis patients (56.3% male, mean age: 60.9 years)	High sclerostin levels are linked to higher risks of cardiovascular events and mortality, but *Dkk-1* is not.
Takashi and Kawanami, 2022 [17]	Review	Not applicable	Sclerostin implicated in insulin resistance and diabetic complications.
He et al., 2021 [18]	POCS	66 hemodialysis patients (57.6% male, mean age: 56.3 years)	Higher serum sclerostin levels were significantly correlated with increased coronary artery calcification and cumulative risk of cardiovascular events, and sclerostin was identified as an independent risk factor for aortic calcification.
Frysz et al., 2022 [19]	POCS	327 CAD patients (64.8% male, mean age: 64.1 years)	Sclerostin levels were associated with increased CAD severity and mortality, cardiovascular disease risk factors like DM, reduced eGFR, higher TGs, and lower HDL cholesterol.
Yüksel et al., 2023 [20]	POCS	175 patients with coronary artery calcification and plaque composition (43.4% male, mean age: 58.4 years)	Sclerostin levels were positively associated with CAC scores and coronary plaque burden, and they predicted MACE over a one-year follow-up period.
Leto et al., 2022 [21]	POCS	174 type 2 diabetes patients (57.5% male, mean age: 56 years)	Sclerostin levels were higher in patients with early vascular stiffness.
González-Salvatierra et al., 2024 [22]	POCS	104 type 2 diabetes patients (60% male, mean age: 62 years)	Sclerostin levels positively correlated with cardiovascular risk based on SCORE2-Diabetes values.
Morena-Carrere et al., 2024 [23]	POCS	425 non-dialysis CKD patients (50.5% male, mean age: 65 years)	Sclerostin contributes to vascular calcification and MACE in CKD patients, but its independent predictive value diminishes after adjusting for confounding factors like diabetes and cardiovascular history.
Garcia-de los Ríos et al., 2022 [24]	POCS	68 women with systemic lupus erythematosus (0% male, mean age: 43.8 years)	A 10-unit rise in sclerostin levels was linked to a 44% higher likelihood of carotid plaque, even after adjusting for other cardiovascular risk factors.
Lee et al., 2024 [25]	Cross-sectional POCS	44 pre-dialysis ESRD patients (58.1% male, median age: 54 years)	Higher sclerostin levels are associated with pulmonary hypertension in pre-dialysis ESRD patients. (estimated pulmonary artery systolic pressure >35 mmHg on echocardiography)
Carrillo-López et al., 2021 [26]	Review	Not applicable	Disrupted Wnt signaling with elevated sclerostin and RANKL/OPG imbalances contribute to vascular calcification and increased cardiovascular risk in CKD.
Ueland et al., 2021 [27]	POCS	106 precapillary PH patients (30% male, mean age: 44 years)	Although Wnt-related proteins such as Wnt5a, DKK3, sFRP3, and WIF1 were associated with sclerostin as part of the broader Wnt signaling pathway, sclerostin itself did not show a direct correlation with increased pulmonary vascular resistance or poor prognosis in precapillary pulmonary hypertension.
Kern et al., 2020 [28]	POCS	205 patients referred for invasive coronary angiography (70.2% male, mean age: 62.9 years)	Although no direct link was found between sclerostin levels and CAD severity, sclerostin levels showed significant correlations with higher hs-CRP, higher *Klotho* protein, higher mean BMI, lower estimated glomerular filtration rate and lower iPTH.
Sanabria-de la Torre et al., 2022 [29]	Scoping review	11 studies, total 2786 patients (male: from 43.87% to 68%, mean age: from 52.5 to 83 years)	The review found mixed results regarding the relationship between elevated sclerostin levels and CVD and mortality, but there appears to be a positive trend in specific populations that suggests an increased risk of CVD and cardiovascular mortality with higher sclerostin levels.

HD, hemodialysis; PD, peritoneal dialysis; eGFR, estimated glomerular filtration rate; TG, triglyceride; ESRD, end-stage renal disease; *Dkk-1*, *Dickkopf-related protein-1*; CVD, cardiovascular disease; MACE, major adverse cardiovascular events; POCS, prospective observational cohort study.

**Table 2:** Sclerostin and cardioprotective role.

Author (Year)	Methodology	Study Characteristics and Sample Size According to Gender	Key Finding
Golledge and Thanigaimani, 2022 [30]	Review article	Mixed (cross-sectional and prospective cohort studies, experimental studies, RCTs, MR studies)	Sclerostin’s role in cardiovascular disease remains controversial, with both protective and detrimental effects reported.
Tobias J.H., 2023 [31]	Review article	Mixed (preclinical studies, RCT, cross-sectional studies)	While experimental studies, clinical trials, and Mendelian randomization studies suggest that sclerostin may have a cardioprotective role, observational studies have shown conflicting results.
Alcalde-Herraiz et al., 2024 [32]	Mendelian Randomization	Meta-analysis (49,568 individuals, 55% male)	Lower genetically predicted sclerostin levels were linked to increased risk of coronary artery disease and type 2 diabetes, along with unfavorable cardiovascular biomarkers such as reduced HDL and elevated triglycerides.
Zhao et al., 2020 [33]	Cross-sectional study	140 CKD patients (stage 3–5, 51.4% male, mean age: 64.0 years)	Serum sclerostin levels increase as renal function declines in CKD patients and are associated with carotid artery atherosclerosis, with a suggested feedback mechanism potentially protecting against atherosclerosis by inhibiting vascular calcification and inflammation.
D’Onofrio et al., 2020 [34]	Review article	Mixed (preclinical studies, RCTS, cross-sectional studies)	The contribution of osteocalcin and sclerostin to the development of cardiovascular disease is still debated, mainly because of the controversial association between their serum levels and clinically significant cardiovascular outcomes.
Rroji et al., 2020 [35]	Review article	Not applicable	Sclerostin exhibited a dual role by promoting vascular calcification while potentially offering protective cardiovascular effects through modulation of the Wnt/β-catenin signaling pathway.
Shalash et al., 2019 [36]	Cross-sectional study	50 patients with diabetes (46% male, mean age: 52.4 years), 20 healthy controls (35% male, mean age: 50.3 years)	T2DM patients have higher serum sclerostin levels than controls, correlating with increased carotid intima-media thickness and subclinical atherosclerosis. This elevation may be a compensatory response to inhibit vascular calcification and maintain vascular homeostasis.
Lin et al., 2020 [37]	Review	Not applicable	Sclerostin has a dual role: promoting insulin resistance in type 2 diabetes and providing anti-calcification effects during cardiovascular disease.
Skrzypczyk et al., 2021 [38]	Cross-sectional study	60 pediatric patients with hypertension (61.7% male, mean age: 15.0 years), 20 healthy controls (45.0% male, mean age: 14.4 years)	Sclerostin levels are not significantly different between hypertensive and normotensive children; inversely associated with blood pressure.
Ibrahim et al., 2021 [39]	Cross-sectional study	273 postmenopausal females with non-obstructive CAD [no CT: 145, CT: 128, mean age: 57.3 (no CT), 61.3 (CT)]	Sclerostin exerts a protective role by reducing arterial stiffness, inhibiting vascular calcification, and potentially mitigating atherosclerosis, thereby influencing the development of coronary tortuosity in aging, hypertensive, and postmenopausal populations.
He et al., 2020 [40]	Prospective cohort study	310 SCAD patients (43.9% male, mean age: 76.1 years) undergoing PCI	High serum sclerostin levels are associated with better MACCE-free survival and overall survival in elderly SCAD patients undergoing PCI.
Yu et al., 2022 [41]	Experimental study	Not applicable	Targeting sclerostin loop3 can maintain cardiovascular protection and promote bone formation.

MR, Mendelian randomization; RCT, randomized controlled trials; CKD, chronic kidney disease; T2DM, type 2 diabetes mellitus; MACCE, main adverse cardiovascular and cerebrovascular events; SCAD, stable coronary artery disease; PCI, percutaneous coronary intervention; CT, coronary tortuosity.

**Table 3:** Sclerostin inhibitors and cardiovascular events.

Author (Year)	Methodology	Study Characteristics and Sample Size According to Gender	Key Finding
Takeuchi Y., 2021 [42]	Review	Mixed (RCTs, pharmacovigilance studies, observational studies…)	While romosozumab is effective in preventing osteoporotic fractures, concerns remain about its potential cardiovascular risks.
Lv et al., 2020 [43]	Systematic review and meta-analysis	6 RCTs (1 exclusively included men, others predominantly included women)	Romosozumab may increase the risk of 4P MACE in elderly men and postmenopausal women.
Vestergaard Kvist et al., 2021 [44]	Pharmacovigilance analysis	Data from FAERS	Romosozumab exhibits a mixed cardiovascular safety profile, with increased reports of major adverse cardiovascular events (MACE) primarily in older, high-risk patients, while its precise cardiovascular impact remains unclear due to potential confounding factors and uncertain biological mechanisms.
Turk et al., 2020 [45]	Nonclinical safety evaluation	Animal studies	Preclinical studies demonstrated that romosozumab has a neutral impact on atherosclerosis, showing no promotion of vascular calcification, atheroprogression, or related inflammatory processes in animal models.
Fixen and Tunoa., 2021 [46]	Review	6 RCTs (1 exclusively included men, others predominantly included women)	Romosozumab has shown increased cardiovascular adverse events in high-risk patients despite unclear biological mechanisms, while sclerostin’s elevated expression in vascular tissues suggests a potential protective role against vascular calcification.
Asadipooya and Weinstock., 2019 [47]	Review	Mixed (RCTs, pharmacovigilance studies, observational studies…)	Romosozumab shows efficacy in increasing bone mineral density and reducing fractures, but it may be associated with an increased risk of cardiovascular events.
Tominaga et al., 2021 [48]	POCS	262 osteoporosis patients (13.36% men, mean age: 77.06 years, 76.72% primary osteoporosis, 23.28% secondary)	Romosozumab effectively increases bone mineral density but has a context-dependent cardiovascular risk profile, with no cardiovascular events reported in this study despite concerns from previous trials.
Cejka D., 2021 [49]	Review	Mixed (experimental studies, clinical trials, cohort studies, meta-analysis, genetic studies)	Romosozumab treatment in chronic kidney disease patients, particularly those on hemodialysis, is linked to manageable hypocalcemia without clear evidence of increased cardiovascular events, though its impact on vascular calcification remains under investigation.
Kawaguchi, 2024 [50]	Perspective and pharmacovigilance report	Post-marketing data from Japan	Japan reported higher rates of MACE linked to romosozumab due to expanded use in older patients with cardiovascular risks.
Seeto et al., 2023 [51]	Systematic review and Bayesian network meta-analysis	136,940 postmenopausal women	Romosozumab ranked higher for cardiovascular adverse events compared to placebo and most osteoporosis treatments, with the exception of certain bisphosphonates and PTH analogues.
Li et al., 2020 [52]	Meta-analysis reanalysis	6 RCTs (1 exclusively included men, others predominantly included women)	Romosozumab showed no increased risk of cardiovascular events when compared to placebo.
Jacob and Paul., 2022 [53]	Commentary	Not applicable	Romosozumab’s inhibition of sclerostin and activation of the WNT-β catenin pathway may increase cardiovascular risk by reducing adipogenesis and promoting free fatty acid deposition in visceral fat and arterial walls.
Lv et al., 2020 [54]	Response to a meta-analysis reanalysis	Not applicable	Further studies with longer-term follow-up are necessary to better understand romosozumab’s safety profiles.
Bovijn et al., 2020 [55]	Meta-analysis and MR analysis	Meta-analysis (4 RCT), MR analysis (biobank, cohort studies)	Romosozumab may increase cardiovascular risk; genetic analysis supports findings.
Hólm et al., 2021 [56]	Commentary	Not applicable	The genetic data weakly link *SOST* inhibition to cardiovascular risk, likely influenced by other genetic factors.
Bovijn et al., 2021 [57]	Response to commentary	Not applicable	Potential cardiovascular risks of romosozumab warrant further investigation.
Masuda et al., 2024 [58]	Population-based cohort Study	49,104 patients (68% female, mean age: 73 years)	No significant difference in MACE between romosozumab and teriparatide.
Cheng et al., 2024 [59]	Systematic review and network meta-analysis of RCTs	16,777 postmenopausal women	No increased cardiovascular mortality or Mace between romosozumab and placebo.
Stokar and Szalat, 2024 [60]	POCS	11,220 patients (50% female, mean age: 72 years)	Romosozumab was associated with fewer 3PMACE compared to PTH analogs.
Wong et al., 2024 [61]	Systematic review and meta-analysis	13,507 patients (95% female)	No difference in cardiovascular events of Asian subgroup with romosozumab versus placebo.
Macrae et al., 2025 [62]	POCS	41 women (mean age: 71 years)	QRISK3 overestimated cardiovascular risk compared to ESC SCORE, leading to wider romosozumab use despite perceived higher CV risk.

MR, Mendelian randomization; RCT, randomized controlled trials. 4P MACE, 4-point major adverse cardiovascular events; FARES, FDA adverse event reporting system. 3PMACE, 3-point major adverse cardiovascular events.

## Data Availability

No datasets were generated or analyzed during the current study.
